# I know where you'll look: an fMRI study of oculomotor intention and a change of motor plan

**DOI:** 10.1186/1744-9081-5-27

**Published:** 2009-07-02

**Authors:** Raimund Kleiser, Christina S Konen, Rüdiger J Seitz, Frank Bremmer

**Affiliations:** 1 Department of Neurology, Heinrich-Heine-University Düsseldorf, Moorenstr.5, 40225 Düsseldorf, Germany; 2 Department of Neurophysics, Philipps-University Marburg, Renthof 7, 35032 Marburg, Germany

## Abstract

**Background:**

Electrophysiological studies in monkeys showed that the intention to perform a saccade and the covert change in motor plan are reflected in the neural activity of the posterior parietal cortex (PPC).

**Methods:**

To investigate whether such covert intentional processes are demonstrable in humans as well we used event related functional MRI. Subjects were instructed to fixate a central target, which changed its color in order to indicate the direction of a subsequent saccade. Unexpectedly for the subjects, the color changed again in half of the trials to instruct a spatially opposite saccade.

**Results:**

The double-cue induced synergistic and prolonged signals in early visual areas, the motion specific visual area V5, PPC, and the supplementary and frontal eye field. At the single subject level it became evident that the PPC split up in two separate subareas. In the posterior region, the signal change correlated with the change in motor plan: activation strongly decreased when the cue instructed an ipsiversive saccade while it strongly increased when it instructed a contraversive saccade. In the anterior region, the signal change was motor related irrespective of the spatial direction of the upcoming saccade.

**Conclusion:**

Thus, the human PPC holds at least two different areas for planning and executing saccadic eye movements.

## Background

Our eyes are continuously on the move, since we perform about three eye movements per second. This operation requires continuous transformation of visual input to motor output and continuous shifts of visual attention since we attend to where we look. The tight linkage between attention and eye movements is reflected in an extensive overlap of cortical networks [1-3]. The merging is especially prominent in the posterior parietal cortex (PPC), which represents one of the anatomical and functional interfaces between sensory and motor systems [4,5]

Electrophysiological studies in non-human primates showed the involvement of a specific region within PPC, i.e. the lateral intraparietal area (LIP) in either visual attention [6-8] or motor intention [9-11]. Beyond the functional role concerning attentional versus intentional processes, the PPC is also known for playing a role in memory related coding of action [12-18].

In view of the functional heterogeneity of the PPC Bracewell used the 'change of motor plan' paradigm to isolate the intention to perform a saccade from attentional processes [19]. The authors trained macaque monkeys to covertly change the saccadic plan indicated by peripheral visual stimuli in sequence. The results showed that alterations in the intention to execute a saccade were reflected in single-unit activity of area LIP.

In the present study, we asked whether such changes in motor plan were detectable in humans as well. To investigate the cortical processes related to motor intention and to a "change of motor plan" paradigm we employed event-related functional magnetic resonance imaging (fMRI). We assumed that the hemodynamic response in relation to the visual cue of the stimulus indicated motor intention, while the hemodynamic response in relation to the onset of the saccade reflected the instantaneous demands of the change of motor plan. We will show that besides the PPC also other areas of the dorsal stream including the motion specific visual area V5, the frontal eye field (FEF), and the supplementary eye field (SEF) dissociate intention from attention related activation.

## Methods

### Subjects

Thirteen healthy right-handed subjects (eight males and five females, mean age 28, range 23 – 34) participated in the study. Handedness was assessed with the 'Edinburgh Handedness Inventory' [20]. All subjects had normal or corrected to normal vision and had no history of neurological or psychiatric diseases. Informed consent was obtained from each subject before the experiment started, which was approved by the 'Ethic committee of the Heinrich-Heine-University Düsseldorf'.

### Experimental protocol

**Figure 1:**
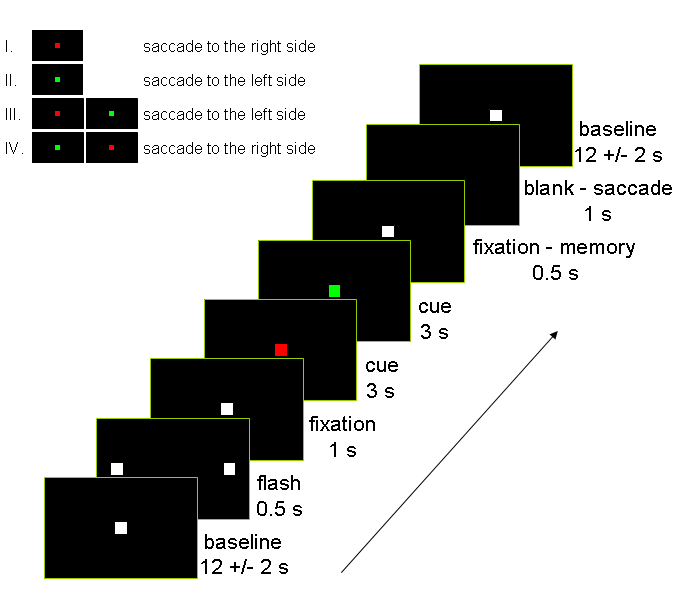
**Experimental task design**. The cue turned either red signaling a saccade to the right side or green signaling a saccade to the left side. Each target position was given by a previously flashed target. The subjects prepared thus a rightward saccade (I. red cue), a leftward saccade (II. green cue), first a right- then a leftward saccade (III. red-green combination), or first a left- then a rightward saccade (IV. green-red combination). The offset of the fixation target indicated the start of the memory saccade.

Figure 1 depicts the schematic illustration of the experimental protocol. Stimuli were displayed on a back-projection screen using an LCD projector (Sony VPL-S500E). The background was continuously black (<1 cd/m^2^). Two points were flashed for 500 ms at minus and plus 10 degrees along the horizontal meridian to indicate (i) the start of each trial and (ii) the possible future location of the memory saccade. Thereafter, a central fixation point (50 cd/m^2^) subtending 1 deg of visual angle was presented for 1 s. Subjects maintained central fixation, while for 3 s the fixation point turned either green (18 cd/m^2^) signaling a saccade to the left or red (10 cd/m^2^) signaling a saccade to the right. Without the advanced knowledge of the subjects, for further 3 s the color of the cue changed again in half of the trials to instruct a spatially opposite saccade. The subjects prepared thus a rightward saccade during the presentation of the red cue, a leftward saccade during the presentation of the green cue, first a right- then a leftward saccade during the red – green combination, and first a left- then a rightward saccade during the green – red combination. Each cue appeared for 3 s. A delay period ensued for 500 ms, followed by the disappearance of the fixation point being the GO-signal for the memory saccade. The delayed saccade task is an important paradigm for investigating the PPC and other saccade related structures [21]. With the use of this paradigm, electrophysiological studies showed distinguishable light sensitive, memory, and saccade related activation in parietal neurons [22].

Finally, the subjects held their gaze at the peripheral location for approximately 1 s before returning back to the center. The baseline consisted of fixation of the central target (12 -/+ 2 s) and was embedded in-between the trials. The intertrial interval provided time for the fMRI signal to return to baseline following each saccade trial. For each subject, there were 500 functional MR scans involving about 100 pseudo-randomly interleaved trials.

Two general linear models were calculated with different time points for aligning the hemodynamic response function. In relation to the first cue we attempted to capture oculomotor intention, while in relation to the onset of the saccade we studied the change of motor plan.

### Data acquisition and analysis

The experiments were performed on a Siemens Magnetom Vision 1.5 T MRI scanner (Erlangen, Germany). Echo planar imaging (EPI) images were acquired continuously with a repetition time (TR) of 4 s and at an image resolution of 3 × 3 × 4.4 mm^3^. The echo time (TE) and effective flip angle were 66 ms and 90 deg, respectively. 30 slices were acquired in axial orientation parallel to the anterior-posterior commissural line. The first 5 scans of all EPI series were discarded from the analysis to minimize magnetic saturation effects. Structural images were acquired using a standard T1-weighted sequence. The data were analyzed using the general linear model for event-related designs of the Statistical Parametric Mapping software package (Wellcome Department of Imaging Neuroscience, London, UK, ).

The functional images were realigned, slice scan time-corrected, and normalized. The transformed images were spatially smoothed with a Gaussian kernel using 10 mm FWHM (full width half maximum). Finally, the functional and anatomical images were co-registered and transformed into Talairach space [23].

In order to allow inferences about the population, the contrast images from all subjects were entered into a group-level random effects analysis (RFX). The resulting statistical maps were thresholded at P < 0.001, uncorrected for multiple comparisons. The time courses were aligned to the onset of the first cue and averaged across subjects. The hemodynamic response to cue onset for each condition was modeled by the synthetic hemodynamic response function with temporal derivatives.

Differences in blood oxygenation level-dependent (BOLD) responses were tested with an analysis of variance (ANOVA) followed by a multiple comparison test (t-test). All t-tests were always 2-tailed. We chose the percent BOLD signal change over time to test for differences between signal amplitudes.

The horizontal eye movements were constantly recorded at 500 Hz during data acquisition ([24] Cambridge Research System, Rochester, UK) and subsequently analysed with Matlab 7 (Mathworks, Inc.). The saccade onset was detected by velocity criterion (three consecutive samples above 10% of peak velocity).

## Results

### Behavioral results

The mean latencies for single-cue trials were 223 ms (87 ms SD) for rightward saccades (red cue) and 217 ms (84 ms SD) for leftward saccades (green cue). The mean latencies for double-cue trials (red-green and green-red) were 223 ms (84 ms SD) and 221 ms (77 ms SD), respectively. Reaction times between the four conditions were not different (ANOVA, P > 0.05). Based on absolute latency values, 8% of the eye movements were considered to be express saccades (70–130 ms), 22% were fast-regular (140–180 ms), and 70% were slow-regular saccades (>190 ms) (for classification see [25]). Errors occurred in less than 5% of the trials and were excluded from the further analysis.

### Functional imaging results

#### Random effects analysis with the first cue as predictor

**Figure 2:**
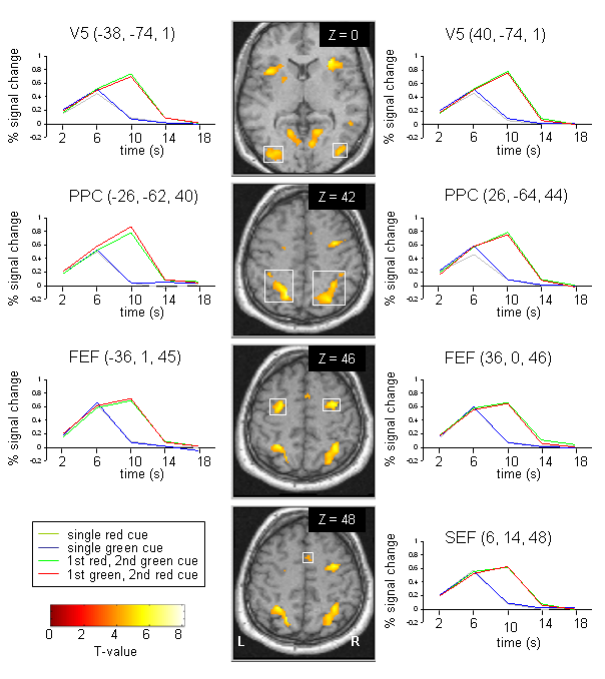
**Percent signal change against time for significant activated regions**. The time courses were aligned to the onset of the first cue and averaged across subjects. Talairach coordinates are given. The gray and blue lines indicate the conditions consisting of the red and green cue, respectively. The green line represents the sequence of first red then green cue, the red line represents the sequence of first green then red cue. PPC = posterior parietal cortex, FEF = frontal eye field, SEF = supplementary eye field, L = left hemisphere, R = right hemisphere.

We calculated the mean fMRI responses for all conditions relative to fixation based on the group-level random effects analysis (F{4,48}, P < 0.001, uncorrected). The results revealed a widespread activation of areas rendering the sensory-motor transformation [26]. Activation of areas along the dorsal pathway and corresponding signal time courses are displayed in Figure 2.

The PPC spreading from the intraparietal sulcus (IPS) to the superior parietal lobule was labeled by an enhanced signal change in case that two cues were successively presented compared to a single-cue (degrees of freedom: 12, P < 0.01, uncorrected). Furthermore, the BOLD response was significantly prolonged during the double-cue as opposed to the single-cue (degrees of freedom: 12, P < 0.05, uncorrected). The motion specific visual area V5 around the inferior temporal sulcus exhibited a similar response pattern. Again, as expected the activation was elevated during the conditions consisting of double-cues as compared to individual cues (degrees of freedom: 12, P < 0.01, uncorrected). The fMRI signal was prolonged for the consecutively presented cues (degrees of freedom: 12, P < 0.01, uncorrected). The BOLD amplitude of the SEF of the dorsomedial frontal lobe and the FEF along the precentral sulcus showed a similar activation pattern (Figure 2). The BOLD response was prolonged when the double cue was presented as compared to the single cue (degrees of freedom: 12, P < 0.01, uncorrected). Note, the comparisons between single and double cue are mainly appropriated control contrasts and also for an entire supplementation.

#### Random effects analysis with onset of saccadic eye movement (GO-signal) as predictor

We also calculated the group-differences in brain activation between the conditions consisting of single- and double-cues at the time of the performance of the saccade. This comparison mainly revealed activation related to the execution rather than the intention to perform a saccade. Therefore, the time courses were aligned to the onset of the saccade which was determined by an offline saccade-detector (see Methods). Note that this point in time occurred about 3 s earlier for the single-cues as compared to the double-cues. Bilateral area V5, PPC, FEF, and SEF showed greater activation during the double- than during the single-cue condition (F{2,24}, P < 0.05, uncorrected). The reversed contrast yielded no significant activation.

### Posterior parietal cortex

**Figure 3:**
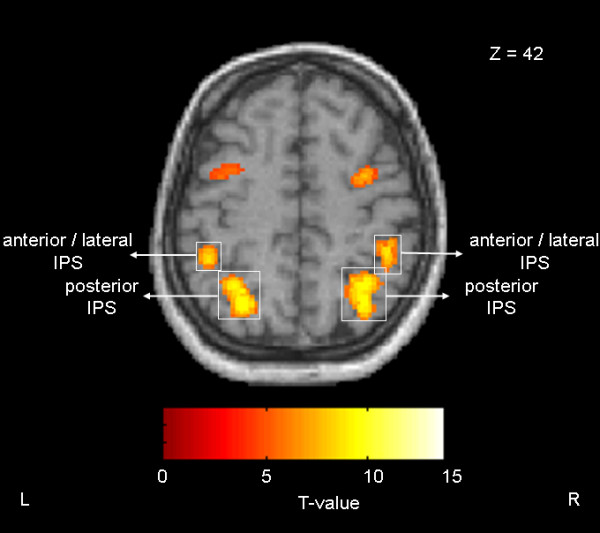
**Activation pattern of a representative subject as a result of the comparison of the double-cue condition relative to baseline**. The areas were significantly activated at P < 0.05 corrected. The figure depicts differential activation between the anterior/lateral and the posterior IPS. IPS = intraparietal sulcus, L = left hemisphere, R = right hemisphere.

The single subject analysis was performed within regions of interest (ROIs). First, a small volume correction with a sphere of 10 mm radius was used according to the coordinates and anatomical landmarks (posterior and anterior/lateral IPS) of our previous study [27]. Second, we selected voxels within each ROI that showed a significant effect for the double-cue condition (P < 0.05 corrected for FWE). Figure 3 shows the parietal activation pattern of a representative subject.

**Figure 4:**
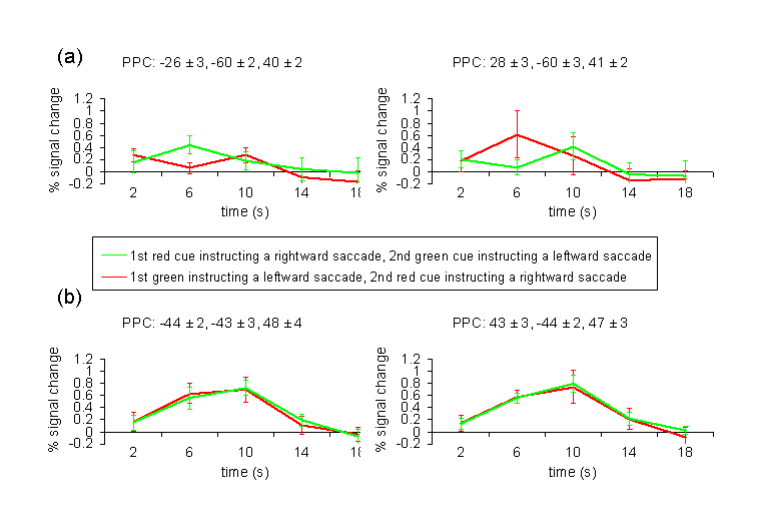
**Percent signal change against time**. The time courses were aligned to the onset of the first cue and averaged across subjects n = 13. (a) Signal time course for the posterior IPS. (b) Signal time course for the anterior/lateral IPS.

Activation within the posterior ROI of the right hemisphere was increased whenever the cue was green and thus instructed a saccade to the left side, while activation was decreased when the cue was red instructing a saccade to the right side. Activation within the posterior ROI of the left hemisphere revealed the reversed pattern of activation. The averaged time courses showed that activity increased whenever the cue instructed a contraversive saccade while it decreased when the cue instructed an ipsiversive saccade (Figure 4a). In order to compare fMRI signals corresponding to the motor plan for contra- and ipsiversive saccades, we calculated the difference between successive time points (TPs). Due to the HRF and the fMRI parameters used in our study (TR = 4 s, see Methods for details), the TP1 corresponded to the fMRI signal change at 2 s, TP2 corresponded to the fMRI signal change at 6 s, and TP3 corresponded to the fMRI signal change at 10 s. Thus, TP2-TP1 showed an increase/decrease during the 1^st ^cue period and TP3-TP2 showed an increase/decrease during the 2^nd ^cue period. In both hemispheres, the percent signal change was greater following a cue signaling a contraversive saccade as compared to a cue signaling an ipsiversive saccade (paired t-test, degrees of freedom: 12, P < 0.05) irrespective of the plan or the changed plan of an upcoming eye movement.

The anterior and more lateral ROI revealed a completely different pattern of activation, i.e. no differences between both cue periods (paired t-test, degrees of freedom: 12, P > 0.05, uncorrected). Irrespective of the indicated direction of the subsequent saccade, the fMRI signal of both hemispheres increased continuously during the successive presentation of the cues reaching its peak activation around the onset of the saccade (Figure 4b). Individual coordinates are given in Table 1.

**Table 1:** Coordinates of activation

	**Posterior IPS**								**Anterior/Lateral IPS**							
	
	Left Hemisphere				Right Hemisphere				Left Hemisphere				Right Hemisphere			
	
Subject	X	Y	Z	t-value	X	Y	Z	t-value	X	Y	Z	t-value	X	Y	Z	t-value
S1	-23	-60	39	6,2	29	-60	40	7,1	-46	-47	46	7,1	41	-45	52	6,7
S2	-24	-62	40	7,1	26	-62	38	5,9	-43	-42	42	5,4	43	-42	46	4,9
S3	-29	-61	39	5,3	33	-64	39	8,9	-46	-46	47	5,9	40	-46	47	6,3
S4	-23	-60	38	6,8	26	-61	41	7,5	-45	-45	45	6,7	39	-48	43	7,9
S5	-22	-63	41	5,8	24	-58	42	7,7	-44	-41	47	7,2	45	-45	49	9,2
S6	-21	-56	40	6,2	28	-57	38	6,7	-41	-42	49	5,3	44	-42	50	8,4
S7	-24	-62	42	5,8	24	-56	43	12,9	-42	-40	40	5,9	42	-41	42	11,2
S8	-28	-60	38	6,4	30	-62	39	7,7	-44	-45	43	10,2	47	-47	48	10,4
S9	-30	-62	44	5,2	26	-56	44	12,2	-45	-47	48	8,1	39	-41	47	6,7
S10	-28	-60	37	10,2	28	-64	42	6,2	-43	-44	49	6,9	42	-43	48	5,9
S11	-23	-58	40	11,2	32	-61	38	5,4	-44	-40	52	7	45	-46	49	6,3
S12	-26	-58	39	8,9	26	-58	42	5,7	-42	-41	55	8,2	46	-42	44	7,4
S13	-32	-58	42	6,7	31	-59	44	7,6	-43	-39	54	6,7	44	-41	42	7,8

peak	-26	-60	40		28	-60	41		-44	-43	48		43	-44	47	

Talairach coordinates and t-values of peak activation in PPC from individual subjects' data (P < 0.05 corrected for multiple comparisons, height threshold t = 4.81).

Taken together, activation of the PPC was divided in two separate subregions with different functional implications. Activation of the posterior part of the IPS was strongly decreased when the cue predicted an ipsiversive saccade and strongly increased when it predicted a contraversive saccade. In contrast, the fMRI signal of the anterior part of the IPS was enhanced during the presentation of the cues and declined after the execution of the saccade irrespective of the direction of the eye movement.

## Discussion

In this study we have shown that the PPC together with visual area V5, the FEF and SEF are involved in covertly changing the plan for saccadic eye movements. Moreover, the PPC comprised a posterior subarea at the IPS related to the ultimate direction of the upcoming saccade and an anterior area at the IPS related to motor intention irrespective of the spatial direction of the upcoming saccade. Thus, we showed a double dissociation of the change of motor plan and oculomotor intention in the PPC lining the IPS.

Admittedly, the study of overt as well as covert motor plans is inevitably accompanied by attentional processes. In our study, however, cues that directed the upcoming saccade were presented foveally. Hence, the direction of the attention was de-coupled from the direction of the upcoming saccade. We therefore are convinced that the observed BOLD activity was not attributed to stimulus-driven attention [28]. On the other hand, the 'motor theory of attention' proposes that saccadic eye movements are always preceded by the corresponding shift of attention to the new location [29]. This shift of attention, however, is definitely coupled with the shift of intention and thus cannot be experimentally separated. In contrast, in our paradigm subjects had to keep their attention at the central location, since the offset of the fixation target was the GO-signal for the saccade. Our on-line oculomotor recordings showed that the subjects did in fact fixate the central stimulus as required. Therefore our results are in line with and further extend Rizzolatti's premotor theory of attention [30]. One may speculate that since the activation amplitude was different in the changing motor plan condition, PPC and V5 were involved in the change of the motor plan and SEF and FEF were more involved in attention.

Area V5 revealed an enhanced and prolonged fMRI signal in case two cues were successively presented as compared to an individual cue. This region is usually implicated in motion analysis [31], but can be activated also by both covert and overt shifts of attention [1]. Yet, the exact role of area V5 in the context of spatial shifts of attention/intention remains unclear. Its activation may reflect an attentional shift across the visual scene [32] or the conveyance of visual information from lower- to higher-level regions [33]. Activation of area V5 could also result from feedback projections of parietal and/or frontal eye fields, which are closely interconnected [33,34]. Our data would favor the latter hypothesis, since the activation patterns of area V5 and PPC were highly similar.

Activation of the PPC was also increased when two cues were presented successively as compared to a single cue. This synergistic effect of activation could arise from the change of motor plan according to the presentation of an additional cue. Kimmig et al. [35] showed that the repeated performance of saccadic eye movements is integrated over time in the fMRI signal. In parallel with this finding, repeated shifts of intention could also have an additive effect on the signal time course. Finally, peak activation of the PPC was temporally prolonged when the first cue was followed by an additional one. The time of peak activation thus reflected the motor component of the actual execution of the saccade. In electrophysiological experiments in monkeys, similar activation patterns are observed in area LIP [36] and in saccade related neurons with buildup activity of the superior colliculus [37]. Area LIP projects directly to the superior colliculus [38,39]. Furthermore, Basso and Wurtz [40,41] demonstrated that an increase of target uncertainty results in decreased activity of superior colliculus neurons. Thus, the activity varies with the probability that a particular saccade will be performed. In our experiment, the introduction of the second cue raised the target certainty from 50% to 100%. The degree of certainty thus might also contribute to the enhanced activation of the PPC during the presentation of the second cue.

The FEF is a cortical area that selects visual targets, allocates attention, and programs saccadic eye movements. FEF is also involved in a neural mechanism that can correct saccade errors before visual afferent or performance monitoring signals can register the error [42,43]. The FEF as well as the SEF exhibited no significant modulation of activation by either condition, except for the prolonged BOLD signal when two cues were presented in sequence. In correspondence with this finding, recent imaging studies showed that the FEF and the SEF-proper are mainly involved in the execution of a saccade [44-46]. Nevertheless, the BOLD signal continuously increased during the cue phase, even though the second cue failed to elicit stronger activation as opposed to the first cue. This rise in activation could be either related to sustained attention or preparation of motor response [47,48].

### Posterior parietal cortex

The most important finding of the present study was that the PPC splits up into two different functional and anatomical modules. This became evident in each individual subject. However, it was obscured in the group mean images most probably due to considerable anatomical and functional variability of the posterior parietal cortex as evident from recent meta-analyses [49,50]. The posterior IPS carried a signal, which seemed to encode the spatial direction of the intended saccade, since it showed a lateralization of activation in the hemisphere opposite to the direction of the saccade. Independent of whether a gaze shift actually occurred, activation strongly decreased when the cue instructed an ipsiversive saccade while it strongly increased when it instructed a contraversive saccade. Accordingly, electrophysiological studies showed that the motor receptive fields of area LIP are mostly restricted to the contralateral visual field [34]. Area LIP in monkeys is clearly situated in the lateral bank of the IPS. It should however be mentioned that it is still a matter of debate which human area constitutes the exact homolog of area LIP in the monkeys [47,49,51-53]. Silver et al. suggested that human LIP might have more subdivisions than monkey LIP [54]. However, the anatomical location of the region as found in our study would rather correspond to area cIPS, which lines the caudal part of the IPS [55]. This area is involved in the generation of saccades [56] and plays a role in attentional and intentional processes [57]. It also corresponds well to coordinates of cIPS described by Shikata et al. [18,58].

In contrast, the anterior and lateral part of the IPS mainly encoded the performance of the saccade *per se*. In the absence of a spatial bias, the peak activation occurred around the actual execution of the saccade. The time course indicated the preparation of the motor response rather than its spatial tuning. According to the meta-analysis of Seitz and Binkofski [55], the anatomical localization of this region would correspond to the putative area LIP in humans. On the other hand it could also correspond to the putative human homolog of area AIP [18]. The anatomical variations found in these studies raise the question about the correspondence between area LIP in man and monkey [57].

## Conclusion

From our point of view, these data rather indicate the existence of multiple parietal eye fields in the human cortex [1,59]. In the present study, we could show that the posterior IPS encoded the spatial direction of the upcoming saccade, whereas the anterior and lateral part of the IPS prepared the motor execution irrespective of the response side. Area LIP in monkeys encodes both characteristics. Beside the spatial coding of intention [11], area LIP also encodes non-spatial, purely motor specific intention [60]. Furthermore, lesion studies in humans support this assumption [61]. It was shown that patients with contralesional neglect were impaired in planning but not executing of contralateral saccades.

Taken together, we followed the 'change of motor plan' paradigm [19] and revealed that at least one subregion of the human PPC encoded the spatial direction of the intention to perform a saccade, whereas another subregion was purely motor specific without directional tuning. Thus, the activity reflected oculomotor intention and the spatial change of motor plan. One might speculate that an online analysis of the parietal signal therefore would allow predicting the human oculomotor behavior.

## Competing interests

The authors declare that they have no competing interests.

## Authors' contributions

RK: design of the study, experimental setup, acquisition and analysis of fMRI data, acquisition and software development for behavioral data, writing MS. CK: acquisition and analysis of fMRI data, writing MS. RS: technical supervision, advisory function, MS revision. FB: design of the study, advisory function, MS revision. All authors read and approved the final manuscript.
